# The Sustainable Development Goals for Water: The Need to Consider Perception, Preference, and Safety

**DOI:** 10.4269/ajtmh.17-0572

**Published:** 2017-08-14

**Authors:** Christine Stauber

**Affiliations:** School of Public Health, Georgia State University, Atlanta, Georgia

In this month’s issue, Brooks et al.^1^ examine the role of organoleptic perception and its association with bacteriologic drinking water quality in the Nyanza Province of western Kenya. This study focuses on two important issues relevant for drinking water in the new Sustainable Development Goals (SDGs)^2^: human sensory perception and the measures of water safety, and the need to be able to measure drinking water quality in resource limited settings. To address the lack of studies, the authors researched how household members’ perception of taste, odor, or overall quality were linked to measured levels of *Escherichia coli* via two detection methods: IDEXX Colilert^™^ Quantitray (Colilert) and the Compartment Bag Test (CBT). The authors also compared the CBT to Colilert to determine its ability to measure microbiologic water quality in the field.

This is the first study to identify an association between organoleptic perceptions of taste or odor and *E. coli* contamination. The study found a statistically significant association between the presence of *E. coli* contamination (> 1 MPN/100 mL) detected via Colilert and those who rated their water as less than excellent for taste or odor. It found similar results for taste or odor perceptions with the CBT, but these associations were not statistically significant. This is an important finding because research on organoleptic properties and bacterial contamination is limited, even more so in developing countries.

The study highlights an important yet simple concept about drinking water quality: consumer instinct is perhaps a good gauge of safety or change in the quality of the source. Water consumers were the first to sound the alarm when they perceived taste, odor, or color changes in West Virginia^3^ and Flint, Michigan^4^ during recent large scale chemical contamination events. However, the role of sensory perception and detection of drinking water contamination is quite complex, both in terms of appropriate methodological approach and interpretation of the results.^5^

Studies on organoleptic perception of drinking water quality have mostly consisted of sophisticated approaches to understanding consumer preference in developed countries.^5^ Few have examined the association between organoleptic perception of water quality and fecal contamination, fewer still in resource limited settings. A cross-sectional survey of 900 households in rural Alabama found no significant associations between organoleptic perceptions of taste or odor and total coliform contamination.^6^ However, total coliforms are not as specific to fecal contamination as *E. coli*, which may account for the different results. In Cambodia, a study found limited statistical significance between perceived safety and the level of *E. coli* contamination.^7^ In this month’s study, Brooks et al.^1^ also found limited association between perceptions of overall quality of the water and *E. coli* contamination, suggesting that individual organoleptic perceptions of taste or odor might be better sentinel surveillance tools than more general perceptions of overall quality or safety.

However imperfect human sensory perception might be, it seems that organoleptic perception is an overlooked area of research for drinking water quality in developing countries. Over the last decade, research on access to water and sanitation has increasingly recognized the role of consumer preference to encourage uptake and usage of household water treatment technologies.^8^ Recent evidence examining latrine use has found that access to a latrine may not imply usage, especially when something else is preferred over the latrine.^9^ Nevertheless, to take advantage of the greatest health benefits of these water and sanitation interventions, consistent and continued use is necessary for the largest impact on health.^8,10^

As progress is made toward achieving the SDGs, consumer perception and preference should be important considerations to include in monitoring and measuring SDG 6.1: “to achieve universal and equitable access to safe and affordable drinking water for all”. If consumer perception and preference is ignored with respect to taste and odor perceptions of drinking water, households may rely on other unsustainable and potentially unsafe sources such as bottled water. In many places, consumers are currently meeting the SDG indicator for water but prefer to pay for bottled water for drinking due to poor perception of piped water supply.^11^

The SDG water and sanitation targets have shifted focus from providing categorical indicators such as improved water to indicators that will require large scale drinking water quality monitoring. In their study this month, Brooks et al.^1^ evaluated the CBT to provide additional information on its potential for monitoring in low-resource settings. This is the first study to compare the CBT and Colilert, and it found moderate quantitative and categorical agreement between them. However, perhaps a more important finding is that agreement was highest (> 90%) between the two methods in detecting samples free from *E. coli* (< 1/100 mL) and those highly contaminated with *E. coli* (> 100/100 mL). Quantitative limitations aside, the CBT may be able to provide sufficient information to promote action and measure progress regarding the bacteriologic safety of drinking water sources. As nations move to adopt and implement the SDGs, the need for scalable and efficient ways to monitor drinking water quality will be tremendous. The CBT is a potentially scalable method, as highlighted in a national demographic and health survey in Peru.^12^

The need to measure the safety of drinking water at a scale brings up substantial questions about the cost and effort required to do so. Delaire et al.^13^ estimated the cost to measure bacterial contamination of piped water supplies in Sub-Saharan Africa and found that microbiological monitoring could be incorporated for a relatively small amount (< 2%) of the national budgets in many countries. Their work and the work of Brooks et al.^1^ provide a better understanding of the opportunities and challenges that will be faced in the implementation of the SDGs. While there are economic and logistical challenges, there are tremendous opportunities to build on the work here in identifying novel and innovative tools to monitor, measure, and assist with improving the safety and quality of drinking water; opportunities we cannot afford to miss.
